# Effects of the Tovertafel^®^ on apathy, social interaction and social activity of people with dementia in long-term inpatient care: results of a non-controlled within-subject-design study

**DOI:** 10.3389/fneur.2024.1455185

**Published:** 2024-11-28

**Authors:** Robert Konrad, Carina Güttler, Natalie Öhl, Christian Heidl, Stefanie Scholz, Christian Bauer

**Affiliations:** ^1^Institute of Rescue, Emergency and Disaster Management, Technical University of Applied Sciences Würzburg-Schweinfurt, Nuremberg, Germany; ^2^Department of Palliative Medicine, University Hospital Erlangen, Friedrich-Alexander-University Erlangen-Nürnberg, Erlangen, Germany; ^3^UMIT TIROL – Private University for Health Sciences and Health Technology, Institute for Nursing Science, Hall in Tirol, Austria; ^4^International Dialog College and Research Institute (IDC), SRH Wilhelm Löhe University of Applied Sciences (SRH WLH), Fürth, Germany

**Keywords:** Tovertafel, dementia, EPWDS, AES, SGDC, apathy, social interaction, social

## Abstract

**Introduction:**

Tovertafel^®^ is a VR-based serious game for dementia care (SGDC) that aims to stimulate residents affected by dementia in nursing homes, promote social and cognitive skills and reduce apathy. The aim of this study is to investigate the effects of using Tovertafel^®^ on apathy, social interaction and social activity of people with dementia (PWD) in long-term inpatient care in Germany.

**Methods:**

In this monocentric intervention study, 25 residents of an inpatient long-term care facility with moderate or severe dementia had two weekly applications of Tovertafel^®^ over a period of 8 weeks. Effects on the residents’ social interaction and activity were recorded before (T1), during (T2) and 1 h after (T3) each intervention using the Engagement of a Person with Dementia Scale (EPWDS). The degree of apathy was assessed using the Apathy Evaluation Scale (AES). Effects of Tovertafel^®^ were examined using a simple repeated measures analysis of variance (ANOVA).

**Results:**

Thirteen residents with moderate (52%) and 12 residents with severe dementia (48%) were included. Results showed that residents’ apathy changed over the course of the trial and was partially reduced. ANOVA revealed significant changes in the positive expression of social participation in the overall group between individual observation times (*p* < 0.001; T1: MW = 2.67, SD = 1.352; T2: MW = 3.66, SD = 1.365; T3: MW = 3.10, SD = 1.300) and a significantly lower negative expression of social participation at T2 (MW = 1.09, SD = 0.358) than at T1 (MW = 1.19, SD = 0.579; *p* = 0.028). There was a significantly higher positive expression of behavioral involvement in the overall group at T3 (MW = 1.17, SD = 0.552) than at T1 (*p* = 0.003) or T2 (*p* = 0.045). Analyses did not find any significant interaction between observation times and degree of dementia.

**Discussion:**

Results of the study show that the use of Tovertafel^®^ over a period of 2 months had significant effects on apathy, social activity and social interaction in people with moderate or severe dementia. Symptoms of apathy could be reduced and social interaction and activity increased. However, due to limitations of the study design and special circumstances of the COVID-19 pandemic situation, findings might be overestimated and must be interpreted with care. Further research is necessary.

## 1 Introduction

Both internationally and nationally, an increasing number of people are affected by dementia. For 2020 WHO and Alzheimer’s Disease International count more than 55 million people worldwide having dementia, with nearly 10 million new cases every year (1, 2). In Germany, at the end of 2021, around 1.8 million people aged 65 or older were living with dementia, which corresponds to 7.3% of people in this age group compared to the total population (3, 4). Dementia is a widely empirically confirmed predictor of nursing home admission earlier or later in life and care (5–8). In Germany, high proportions of people with dementia (PWD) of up to 70% are cared for in nursing homes (9, 10). Dementia is associated with a progressive decline in cognitive and physical abilities as well as a gradual decline in verbal and non-verbal communication skills. Often, PWD in care homes spend much of their time alone with little opportunity to participate in meaningful or stimulating activities (11), resulting in negative consequences for the course of the disease and the quality of life of those affected (11).

The identification, provision and evaluation of stimulating activities for PWD that are associated with positive effects on functional and cognitive abilities as well as quality of life and social participation represent an important field of research for evidence-based care design for PWD. In recent years, an increasing number of psychosocial individual and group interventions for PWD have been developed and studied that use games (*serious games for dementia care*, SGDC) to achieve therapeutic effects in the context of dementia (12). SGDC can be seen as a subset of serious games for health. Serious games in general are games that are not primarily designed for entertainment, but for a specific function such as professional training, education, sensitization (13) and, in the case of serious games for health, also for the purpose of therapy (14). Regarding SGDC, most analog games, such as board games, but also electronic games (video games), are aimed at promoting cognitive skills.

Tovertafel^®^ is a VR-based SGDC available since 2015 that combines visual effects with interactive components and aims to stimulate nursing home residents affected by moderate to severe dementia and reduce apathy in its original development goal (15, 16). Based on interactive projection technology (consisting of a projector, infrared sensors, loudspeakers and a central processor), the Tovertafel^®^ simulates everyday objects such as flowers or leaves, with which the players can interact by moving their hands or arms and which are intended to increase their willingness to be active and move (12, 17). The Tovertafel^®^ is installed in care homes on the ceiling above a table in a group room. There are currently 37 games for the Tovertafel^®^ that are designed to promote cognitive, physical, sensory or social skills and activities in five levels (18).

The present study specifically focused on the Tovertafel^®^ due to its widespread availability, with existing literature suggesting its efficacy. Studies that have already been conducted on the effect of the Tovertafel^®^ show that the games can contribute to the well-being of older PWD (16, 19). To date, the effects on apathy and quality of life of PWD in nursing homes have been increasingly investigated (18). Initial trial experiences from a Dutch, 5-day small-scale intervention study with a sample of PWD (*n* = 6) evaluated the use and effects of the Tovertafel^®^ on apathetic behavior (15). Compared to organized coffee drinking and sitting together in the common room without organized activity, observing caregivers reported a reduction in apathy and sadness as well as an increase in physical activity. There was also evidence of an improvement in satisfaction and a reduction in anger and anxiety (15). Another Dutch quasi-experimental study on the effects of playing with the Tovertafel^®^ on the quality of life of PWD (*n* = 34) showed low to medium significant, short-term improvements in individual dimensions of dementia-specific quality of life (19) for playing for 15 min over five consecutive working days, which was recorded using the QUALIDEM external assessment tool (20).

The outlined monocentric intervention study “Effects of the Tovertafel^®^ on apathy, social interaction and social activity of PWD through use in long-term inpatient care (Entertain)” aims to expand the empirical evidence base on the effects of the Tovertafel^®^ with trial experiences from Germany, focusing on apathy, social interaction and social activity of PWD in long-term inpatient care. The study thus contributes to the improvement of evidence-based care for PWD in nursing homes and to the selection of important psychosocial interventions based on SGDC.

## 2 Materials and methods

### 2.1 Question

The study aimed to investigate the effects of using the Tovertafel^®^ on apathy, social interaction and social activity of PWD in long-term inpatient care in Germany.

The following research questions and hypotheses were addressed:

1. Does the use of the Tovertafel^®^ change the social interaction of PWD in an inpatient long-term care facility?
2. Does the use of the Tovertafel^®^ change the extent of apathy among PWD in a long-term inpatient care facility?

*H1*: The use of the Tovertafel^®^ leads to positive changes in PWD the extent of their social interaction.

*H2*: The use of the Tovertafel^®^ leads to positive changes in PWD to the extent of their apathy.

*H3*: The use of the Tovertafel^®^ leads to positive changes in PWD the extent of their social activity.

### 2.2 Ethics statement

The study concept was submitted to the Ethics Committee of the German Society for Nursing Sciences (DGP) for review and a positive clearing (application no. 21–034) was obtained. The study was also registered in the German Register for Clinical Studies^1^.

### 2.3 Study design

The monocentric intervention study was conducted as part of the project “Pflegepraxiszentrum Nürnberg (Nursing Practice Center Nuremberg) – funding number: 16SV7899” funded by the Federal Ministry of Education and Research (BMBF). The data collection took place in a care facility for people with dementia in Nuremberg. The access restrictions in the context of the COVID-19 pandemic with subsequent bans on entering care and outpatient medical facilities and the resulting complete cessation of social care services made it necessary to review the study concept. After evaluating the data collection options and framework conditions that could be implemented under COVID-19 conditions, this facility for people with dementia proved to be suitable for the Tovertafel^®^ trial in inpatient long-term care. In this facility, it was possible to implement regular use of the Tovertafel^®^ in all four living areas in accordance with the hygiene and contact regulations required by COVID-19. However, due to COVID-19 restrictions and the limitations set by already scarce and overloaded resources in the care facility caused by the pandemic a simple as possible but still feasible study design had to be chosen. Hence, as a compromise between research interests and the demands of the care facility, the study design did not include the implementation of control interventions such as group interactions without Tovertafel^®^ or conventional games. However, as the study primarily aimed to investigate the effects of Tovertafel^®^ in general and not in comparison, the omission of control interventions seemed appropriate.

The study aimed on observing practical application and effects, which is why the testing did not take place under ideal or laboratory conditions, but in a natural setting under real conditions in order to explicitly record the social behavior of the study participants. As people with dementia represent a vulnerable target group, ethical considerations were necessary with regard to the choice of study design. The study conducted was a non-randomized intervention study with a “within-subject” design. In this type of longitudinal study, the effects on the study participants are measured several times over a fixed period of time in order to evaluate changes over time. The participants act as controls for themselves (21).

**Figure 1:**
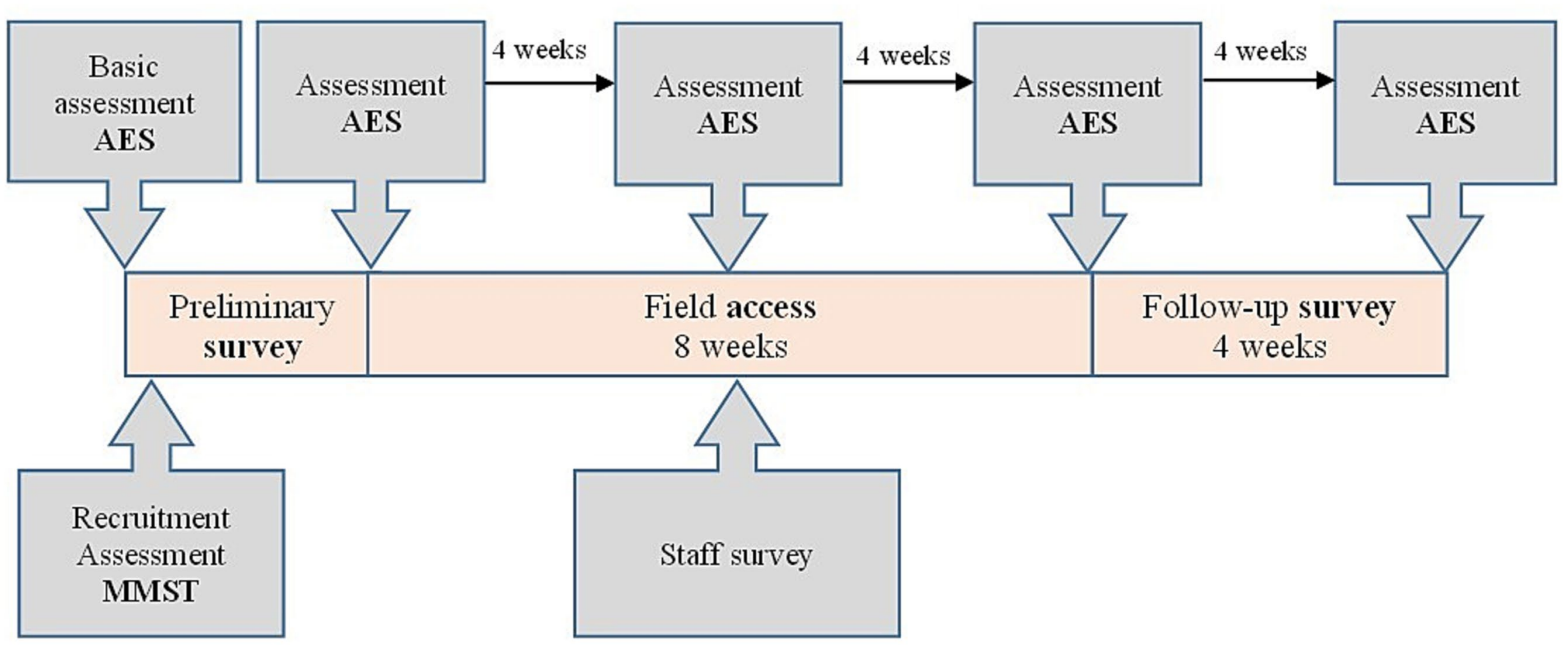
Survey overview of the individual data over time.

The total duration of the study was 17 months. The survey period comprised 8 weeks. In addition, there were 4 weeks of pre-survey preparation for recruitment and 4 weeks of post-survey after field access (Figure 1). The selected study participants took part in 12.64 ± 7.20 applications of the Tovertafel^®^ over a period of 8 weeks (field test), whereby the effects on the social interaction of the study participants were recorded before (T1), during (T2) and 1 h after each intervention (T3) using the Engagement of a Person with Dementia Scale (EPWDS). In addition, the degree of apathy was recorded using the Apathy Evaluation Scale (AES) at five points in time during the study period (Figure 1). The primary outcome objective of the study was the change in social interaction through the use of the Tovertafel^®^, which was assessed before, during and after the use of the Tovertafel^®^ by applying the EPWDS. The use of the survey instruments, the data quality and the analyzability of the data were verified in a pre-test (22) prior to the intervention. A setting close to the trial and a comparable study population were selected for this purpose.

Based on the assessment model of therapeutic effectiveness of SGDC, this study focuses on the measurement of effectiveness through the professional assessments of the nursing staff in conjunction with the test results of the participants. Possible physiological measurements were not relevant to the research questions.

### 2.4 Survey methodology

In accordance with the “within-subject” design, the study participants were observed repeatedly over a fixed period of time. Due to the relatively short-term nature of the effects, the study participants were assessed at three different points in time. This enables a progression analysis for each study participant over the entire survey period and thus an assessment on an individual basis. There was no control group. The assessment was conducted by researchers who had received prior training on the rating scales. On each ward, the same two observers alternated to assess all individuals within an intervention. Being aware that the impressions of the researchers are still subjective, an attempt was made to carry out the survey as consistently as possible.

Data collection in the form of interviews or the completion of questionnaires by the study participants was not possible due to dementia. The severity of dementia was assessed using the Mini-Mental Status Test (MMST) (23), the results of which formed the inclusion (moderate dementia, MMST score from 17 to 10 points and severe dementia, MMST score less than 10 points) and exclusion criteria (all others) for the study project and the care facility. The effects of the Tovertafel^®^ intervention on the social interaction, apathy and social activity of the study participants were recorded using the EPWDS (11) in conjunction with the AES (24) for the main data collection. The EPWDS uses ten questions on a five-point Likert scale to assess the participation of PWD in five dimensions (affective, visual, verbal, behavioral and social participation). Within each dimension, one positive and one negative question is recorded. As the EPWDS was only available in English, it was translated into German by the University of Bremen after obtaining permission for translation from the author. This was followed by repeated translation and retranslation with a test and validation phase. Subsequently, the questionnaire was digitized in the survey tool “Evasys” (version 9.1) (25) for data collection. Since the EPWDS does not include apathy, the AES was also used as a pure apathy assessment tool for data collection for a more detailed assessment of the effects of the use of the Tovertafel^®^ on the apathy of the study participants. This was collected independently of the EPWDS every 4 weeks (Figure 1). The AES was available in a German version and, with its 18 questions, enables apathy to be recorded based on a four-point Likert scale.

### 2.5 Study population

The participants in the intervention study were residents of a care facility for people with dementia. Only people with moderate dementia (MMST score: 17 to 10 points) or severe dementia (MMST score: less than 10 points) were included in the study. The presence of mild dementia was defined as an exclusion criterion for participation in the study, as this does not have a significant impact on quality of life. Further exclusion criteria were the presence of mental stress (e.g., disorientation) as well as blindness or visual problems. Also study participants were not allowed to participate in the Tovertafel^®^ intervention on a daily basis if they experienced acute pain, restlessness or challenging behavior. This was decided immediately before the start of the intervention by the responsible on-duty nursing staff based on their knowledge and assessments of the residents. Daily exclusion of participants during the trial was not necessary. Incomplete datasets from any observation day were excluded from the evaluation.

The study population obtained resulted from an active recruitment process aimed at in the achievement of a high level of willingness to participate voluntarily and on an ongoing basis. The pre-selection of participants was carried out by nursing staff using the MMST basic assessment and the listed inclusion and exclusion criteria. In a two-stage identification process, the participants were first selected by the nursing staff or residential unit managers based on their previous use of social activities and biographical work and then asked personally whether they would be willing to participate.

The participants and their legal representatives were provided with comprehensive information about the research project, the effort involved and the potential benefits and risks by the caregivers. Since the ability to act in decision-making situations can vary in people with dementia or is not always possible in a self-determined manner, the relatives and legal guardians were consulted and signed the information sheets in writing. An information event for relatives and caregivers was also held at the facility, and the project staff were available to clarify any unanswered questions. In order to promote the autonomy of the participants and ensure that participation was voluntary, sufficient time for reflection was granted for the consent to participate in the study. For residents of the care facility who no longer wished to participate in the study from the outset or during the course of the trial, there were no disadvantages in the context of day-to-day care.

The participants, their relatives or legal guardians could withdraw their consent to participate in the study at any time and without giving reasons and decide on the use of the data collected up to that point.

As a result of the described active recruiting process a total of *n* = 25 people took part in the study (seven people in one living area and six people in each of three other living areas).

### 2.6 Implementation of the intervention

The intervention took place in four residential areas, each in a play and social room. Each intervention session usually consisted of six participants from the respective living area. The groups and participants from the different living areas were not mixed during the intervention. Each living area had its own Tovertafel^®^ and a dedicated support assistant. The intervention was carried out twice a week, exclusively in the morning, in order to minimize the risk of absenteeism due to competing activities. This ensured that the data collection was carried out exclusively by the person who accompanied the Tovertafel^®^ intervention. The total playing time of the Tovertafel^®^ was between 30 and 60 min (41 ± 9.65 min), depending on the daily form of the participants.

Each Tovertafel^®^ game is associated with a level of play (levels 1–5), with a higher level of play representing a higher degree of cognitive challenge. The manufacturer provides a brief description of each game, describing the physical, social, sensory and cognitive effects of each game and giving suggestions on how to play it.

Depending on the composition of the participants, 3–4 games were played per intervention session for moderate dementia or 2–3 games for severe dementia. The level of play was determined in each group at the first session and maintained or attempted to be increased during the course of the study. As the clinical picture and symptom behavior in dementia can be very heterogeneous, not all Tovertafel^®^ games were suitable for all participants and all stages of dementia. Social care staff decided on a daily basis and individually which games were to be used and documented the game selection and game level for each group. The selection always included games that were geared toward the abilities of the weakest participant in the sense of a goal-oriented activity.

Games to promote cognition (e.g., word guessing, rummy, hobby pairs), sensory skills (birthday cake, flower, soap bubble) and physical activity (soccer, vegetable garden, silverware) were selected as suitable and applicable games for the study.

Playing with the Tovertafel^®^ was mainly the responsibility of social care staff, who had already been familiar with the proper use of the Tovertafel^®^ in the facility since it was purchased in 2019. This also included, for example, providing chairs and the table purchased for this purpose, as well as creating a pleasant playing environment (e.g., creating good lighting conditions, eliminating background noise) before the intervention with the Tovertafel^®^ began in the living areas. As soon as the Tovertafel^®^ was ready for use, the staff approached each individual participant directly to inform them about the planned social activity. After a successful direct approach, the participants were accompanied to the Tovertafel^®^ unless they objected to participating on the day. The Tovertafel^®^ environment was always barrier-free so that participants with limited mobility could also take part. With a start-up phase of 10 min after the start of the game, the social care staff were able to gain the residents’ attention and interest in the game through empathic and appreciative communication (both verbal and non-verbal), motivate them to participate, guide them to start the game if necessary (hand guidance, tapping on the table) and thus sensitize the participants sufficiently for the activity.

Participation in the activity was always voluntary and could be discontinued at the discretion of the participants or the social care assistants, as well as for personal reasons without the need for an explanation. Participants who discontinued the activity were given the opportunity to withdraw.

### 2.7 Observation phase

The participants were observed by several project staff and employees of the care facility over the course of a game sequence with the Tovertafel^®^, but for a minimum of 10 and a maximum of 15 min. Using the EPWDS observation tool, the observations were documented in the dimensions of affective participation, visual participation, verbal participation, behavioral participation and social participation.

**Figure 2:**
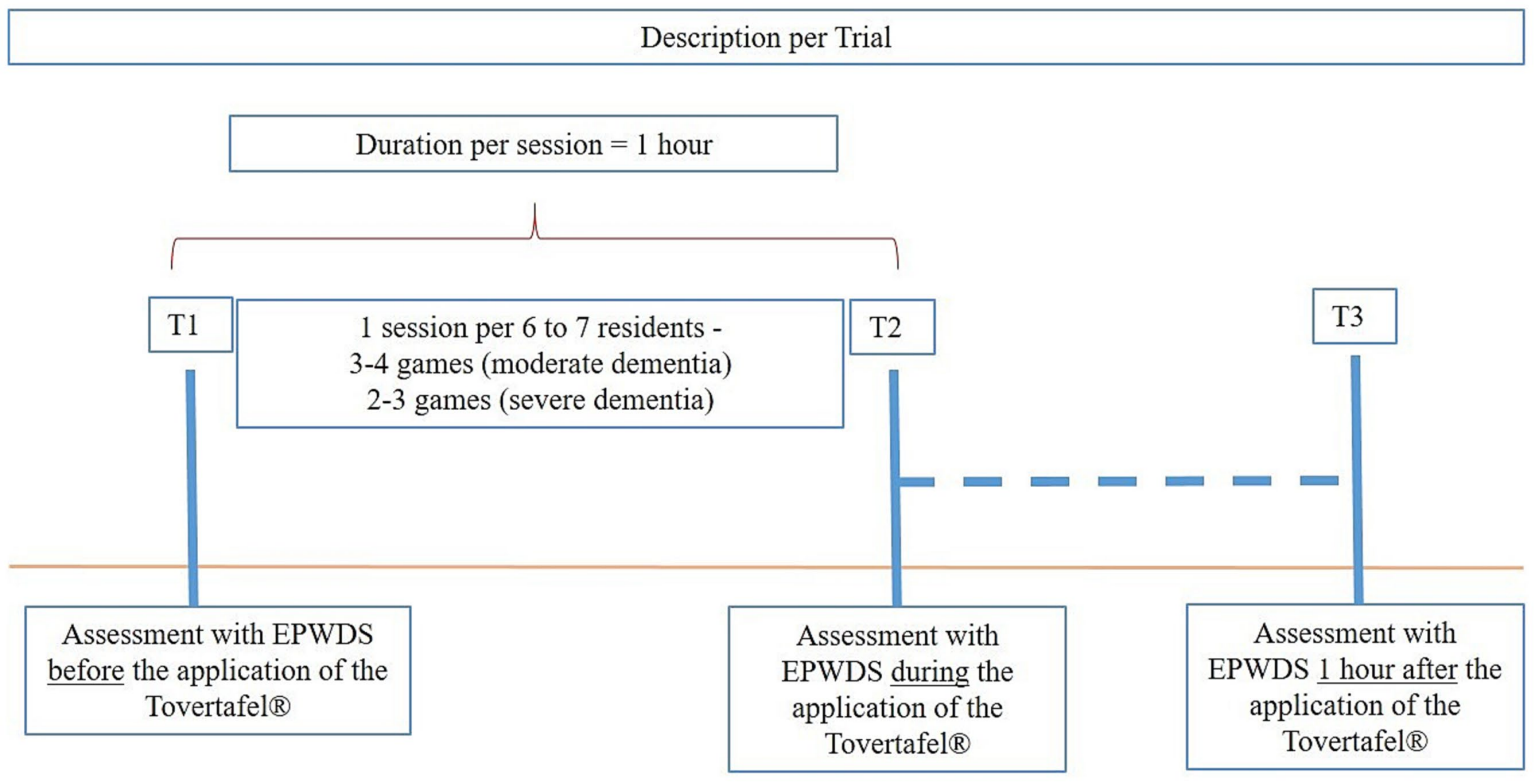
Overview of data collection per Tovertafel^®^ – Application.

There were three observation points (Figure 2) per study participant within the intervention session.

1. Assessment before using the Tovertafel^®^ (T1), during the breakfast control activity
2. Evaluation during the application of the Tovertafel^®^ (T2) and
3. Assessment one hour after using the Tovertafel^®^ (T3), during the lunch control activity

### 2.8 Data evaluation

The data was analyzed using the “within-subject” design. In this case, several measured values are available from the study participants, which leads to a violation of the independence of the observations and had to be taken into account when analyzing the data.

The statistical analysis of the observation results was carried out using SPSS Statistics Version 28 (26). All questionnaires were analyzed descriptively and in relation to the hypotheses and questions. In the data evaluation, a descriptive analysis of the study participants and the four living areas was carried out and the variables were described as frequency (%). In addition, a descriptive analysis of social and behavioral participation was carried out by comparing the mean values of the residents at observation time T1. The primary outcome was the evaluation of the short-term effects of the Tovertafel^®^ using the Hausmann specification test for model validation (FE/RE). A simple analysis of variance with repeated measures (ANOVA) was performed by dividing the participants into groups according to moderate and severe dementia to determine differences between the groups and the test of intersubject effects as logistic panel regression models. In the case of significant ANOVA main effects, post-hoc tests were used. If necessary, the Greenhouse–Geisser correction for non-sphericity was applied. Partial eta squares (η^2^) were used as a measure of effect size, with limits at 0.01 (small effect) and 0.14 (large effect) (27). The significance level was set at *α* < 0.05.

For missing values, the value 0 (not applicable) was defined in the SPSS dataset. The deviation of the mean values in the ANOVA can be explained by the fact that only cases with a valid value in all three variables are included.

A case number estimation was carried out as part of the study planning. The target power of 80 percent was met with the planning of 16 sessions and 24 participants and a minimum medium expected effect size of the intervention (Cohen’s *d* > 0.3).

## 3 Results

### 3.1 Description of the study participants

The characteristics and composition of the trial participants are described in Table 1.

**Table 1:** Descriptive data overview of the study population.

Age (*n* = 25) (MW: mean, Md: median)				
MW; (Md)	83.9; (83.5)			
Range	65–98			
Gender (*n* = 25)				
Female (f)	18 (72%)			
Male (m)	7 (28%)			
Degree of dementia (*n* = 25)				
Moderate dementia	13 (52%)			
Severe dementia	12 (48%)			
Distribution of degrees of dementia by gender (*n* = 25)				
Moderate dementia	w: 9	m: 4		
Severe dementia	w: 9	m: 3		
Degree of care (*n* = 25)				
Care level 2	5 (20%)			
Care level 3	11 (44%)			
Care level 4	6 (24%)			
Care level 5	3 (12%)			
Number of assessments/data sets per living area (*n* = 337)				
Living area 1	95 (28.2%)			
Living area 2	90 (26.7%)			
Living area 3	71 (21.1%)			
Living area 4	81 (24.0%)			
Distribution of degrees of dementia per living area (WB) (*n* = 25)				
	WB 1	WB 2	WB 3	WB4
Moderate dementia	2	6	3	2
Severe dementia	5	0	3	4
Total	7	6	6	6

The descriptive presentation of the data overview of the study population shows that a total of *n* = 25 people were included in the study. The age of the participants varied between 65 and 98 years. The mean is 83.9 and the median is 83.5 years. The participants were divided into 18 (72%) females and seven (28%) males (*n* = 25).

The degree of dementia of the people involved is divided into moderate dementia with 13 people (52%) and severe dementia with twelve (48%) participants. The participants with moderate dementia are divided into nine female and four male residents and the participants with severe dementia are divided into nine female and three male residents.

The care level of the residents is broken down into care level 2 with five people (20%), care level 3 with eleven participants (44%), care level 4 with six participants (24%) and care level 5 with three people (12%).

The number of assessments/records per residential area (*n* = 337) is broken down into residential area 1 with 95 (28.2%) observations, residential area 2 with 90 (26.7%) observations, residential area 3 with 71 (21.1%) observations and residential area 4 with 81 (24.0%) observations.

Finally, the distribution of dementia levels per living area (*n* = 25) shows two participants with moderate dementia in living area 1, six participants in living area 2, three participants in living area 3 and two participants in living area 4. The distribution of dementia levels per living area (*n* = 25) shows five participants with severe dementia in living area 1, no participants in living area 2, three participants in living area 3 and two participants in living area 4.

In the overall overview of the distribution of dementia levels per residential area (*n* = 25), there are seven participants in residential area 1, six participants in residential area 2, six participants in residential area 3 and six participants in residential area 4.

The ongoing changes in social interaction (H1), for example in the form of increased interaction with other people or distraction of other people, are presented below. Two evaluations were carried out to show the changes in the overall group of study participants and according to the degree of dementia of the residents. In the following, the changes in the overall group of participants are discussed first, followed by the changes in residents with severe or moderate dementia.

### 3.2 Changes in social interaction (H1)

#### 3.2.1 Positive expression (e.g., using the activity to encourage other people to interact or as a means of communication to interact with other people)

The positive expression of social participation was lowest in the overall group at observation time T1 (MW = 2.67; SD = 1.352), was highest at time T2 (MW = 3.66; SD = 1.365) and decreased again at observation time T3 (MW = 3.10; SD = 1.300) (Table 2).

**Table 2:** Positive expression of social participation by degree of dementia and time of observation.

	Degree of dementia (MMST)	Mean value	Standard deviation	*n*
Social participation (positive characteristics) – T1	Severe dementia	2.53	1.325	118
	Moderate dementia	2.80	1.368	126
	Total	2.67	1.352	244
Social participation (positive characteristics) – T2	Severe dementia	3.36	1.381	118
	Moderate dementia	3.95	1.289	126
	Total	3.66	1.365	244
Social participation (positive characteristics) – T3	Severe dementia	2.88	1.322	118
	Moderate dementia	3.31	1.249	126
	Total	3.10	1.300	244

A repeated measures ANOVA with the assumption of sphericity found that the positive expression of social participation in the overall group differed statistically significantly between the observation times (*F*(2, 486) = 61.546; *p* < 0.001; partial *η*^2^ = 0.202). A post-hoc test showed a significantly higher positive expression of social participation at observation time T2 than at time T1 (MW_Diff_ = −0.996; 95%-CI [0.769, 1.223], *p* < 0.001), a significantly lower positive expression at observation time T3 than at time T2 (MW_Diff_ = 0.561; 95%-CI [0.361, 0.761]; *p* < 0.001) and a significantly higher positive expression of social participation at observation time T3 than at time T1 (MW_Diff_ = −0.434; 95%-CI [0.212, 0.657]; *p* < 0.001).

For participants with severe dementia, the positive expression of social participation was lowest at observation time T1 (MW = 2.53; SD = 1.325), highest at time T2 (MW = 3.36; SD = 1.381) and decreased again at observation time T3 (MW = 2.88; SD = 1.322). This trend was also visible for residents with moderate dementia between the observation times (T1: MW = 2.80; SD = 1.368; T2: MW = 3.95; SD = 1.289; T3: MW = 3.31; SD = 1.249) (Table 2).

A repeated measures ANOVA with assumption of sphericity showed no significant interaction effect between the observation time points and the MMST groups (*F*(2, 484) = 1.586; *p* = 0.206; partial *η*^2^ = 0.007).

Furthermore, the mean values of the characteristics of the individual residents and their dispersion at the observation time T1 were recorded in order to check whether learning effects (carry-over effects) (28) occurred among residents during the observations and whether they therefore already knew over time what to expect from the Tovertafel^®^ application. The mean comparison of the positive characteristics of social participation is shown below in Table 3.

**Table 3:** Mean value comparison of the positive expression of social participation at time T1.

Degree of dementia (MMST)	Mean value	Median	Standard deviation	Variance (s^2^)
Severe dementia	2.49	2.00	1.358	1.843
Moderate dementia	2.72	2.00	1.361	1.851
Total	2.62	2.00	1.367	1.868

The mean value of the positive expression of social participation at observation time T1 was 2.49 for residents with severe dementia, 2.72 for residents with moderate dementia and 2.62 for the overall group. The median in all groups was 2.00. The standard deviation and variance were greatest in the overall group (SD = 1.367; *s*^2^ = 1.868) and greater in residents with moderate dementia (SD = 1.361; *s*^2^ = 1.851) than in residents with severe dementia (SD = 1.358; *s*^2^ = 1.843) (Table 3).

#### 3.2.2 Negative expression (e.g., distracting or disturbing other people in response to the activity)

The negative expression of social participation was highest in the overall group at observation time T1 (MW = 1.19; SD = 0.579), decreased at time T2 (MW = 1.09; SD = 0.358) and increased again at observation time T3 (MW = 1.17; SD = 0.552) (Table 4).

**Table 4:** Negative expression of social participation by degree of dementia and time of observation.

	Degree of dementia (MMST)	Mean value	Standard deviation	*n*
Social participation (negative expression) – T1	Severe dementia	1.29	0.693	118
	Moderate dementia	1.10	0.428	126
	Total	1.19	0.579	244
Social participation (negative expression) – T2	Severe dementia	1.15	0.483	118
	Moderate dementia	1.02	0.153	126
	Total	1.09	0.358	244
Social participation (negative expression) – T3	Severe dementia	1.25	0.679	118
	Moderate dementia	1.10	0.388	126
	Total	1.17	0.552	244

A repeated measures ANOVA with Huynh-Feldt correction showed a significant difference in the negative expression of social participation in the overall group between the observation times (*F*(1.900, 461.821) = 3.421; *p* = 0.036; partial *η*^2^ = 0.014). Using a post-hoc test, a significantly lower negative expression of social participation was found at observation time T2 than at observation time T1 (MW_Diff_ = 0.102; 95%-CI [0.008, 0.197]; *p* = 0.028).

The negative expression of social participation was highest among residents with severe dementia at observation time T1 (MW = 1.29; SD = 0.693), decreased at time T2 (MW = 1.15; SD = 0.483) and increased again at observation time T3 (MW = 1.25; SD = 0.679). In participants with moderate dementia, this was highest at observation time T1 (MW = 1.10; SD = 0.428), decreased at time T2 (MW = 1.02; SD = 0.153) and returned to the initial mean value at observation time T3 (MW = 1.10; SD = 0.388) (Table 4).

A repeated-measures ANOVA with Huynh-Feldt correction did not detect a significant interaction effect between the observation time points and the MMST groups (*F*(1.908, 461.737) = 0.309; *p* = 0.724; partial *η*^2^ = 0.001).

The mean value of the negative expression of social participation at observation time T1 was 1.29 for residents with severe dementia, 1.09 for residents with moderate dementia and 1.19 in the overall group. The median in all groups was 1.00. The standard deviation and variance at observation time T1 were greatest for residents with severe dementia (SD = 0.710, *s*^2^ = 0.505) and greater in the overall group (SD = 0.578; *s*^2^ = 0.334) than for residents with moderate dementia (SD = 0.401; *s*^2^ = 0.161) (Table 5).

**Table 5:** Mean value comparison of the negative expression of social participation at time T1.

Degree of dementia (MMST)	Mean value	Median	Standard deviation	Variance (s^2^)
Severe dementia	1.29	1.00	0.710	0.505
Moderate dementia	1.09	1.00	0.401	0.161
Total	1.19	1.00	0.578	0.334

### 3.3 Changes in apathy (H2)

**Figure 3:**
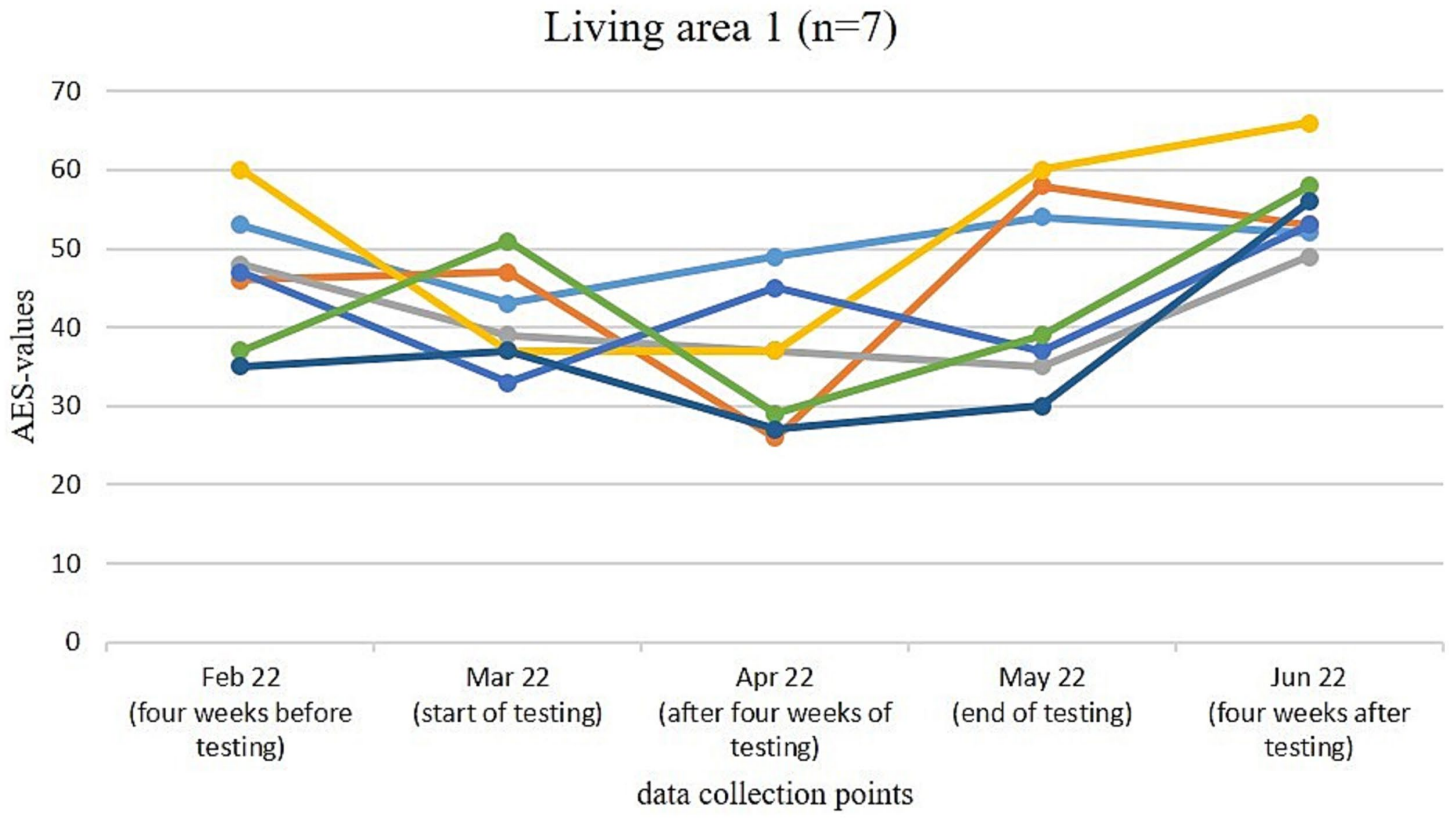
Change in the AES values of residents in living area 1.

**Figure 4:**
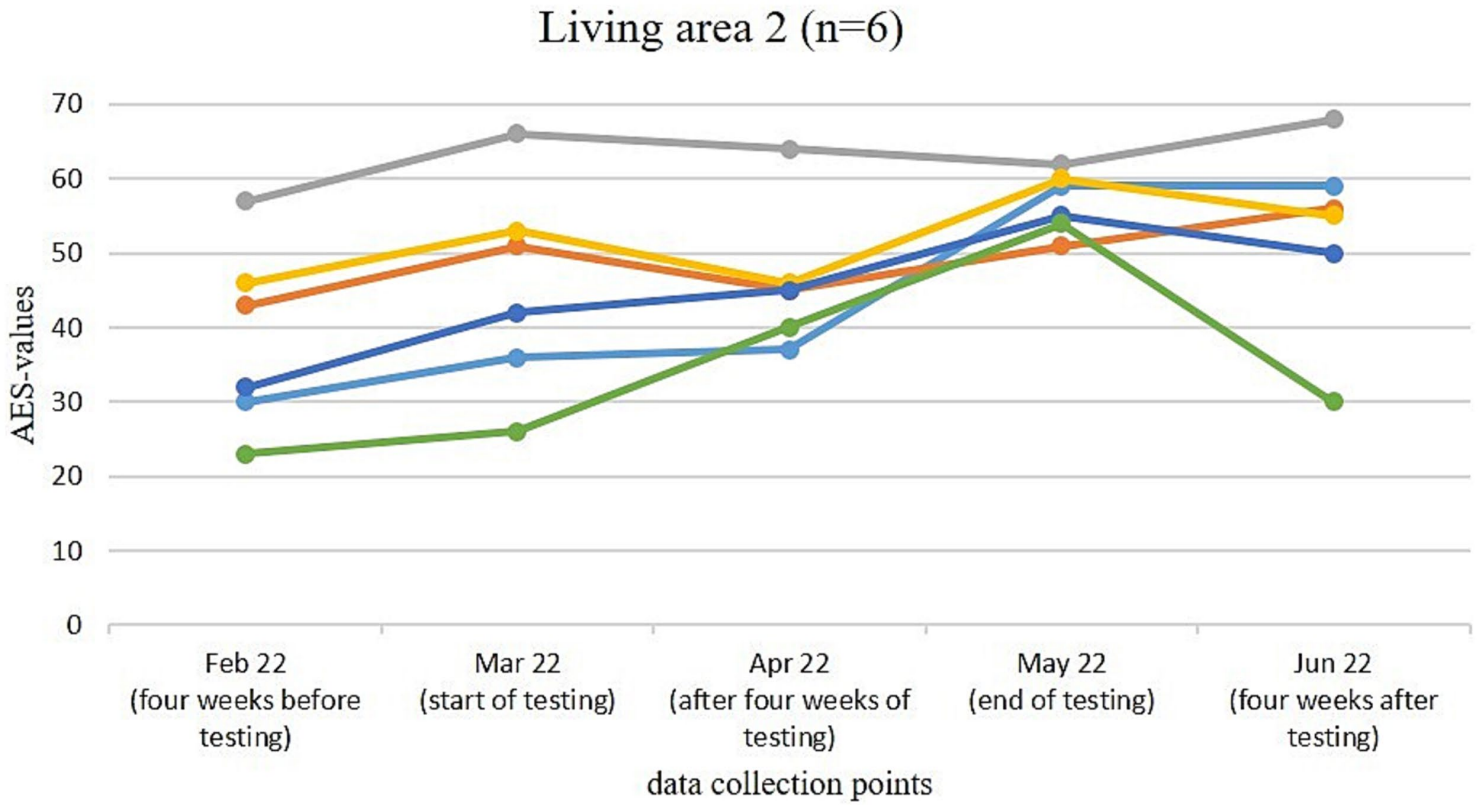
Change in the AES values of the residents in living area 2.

**Figure 5:**
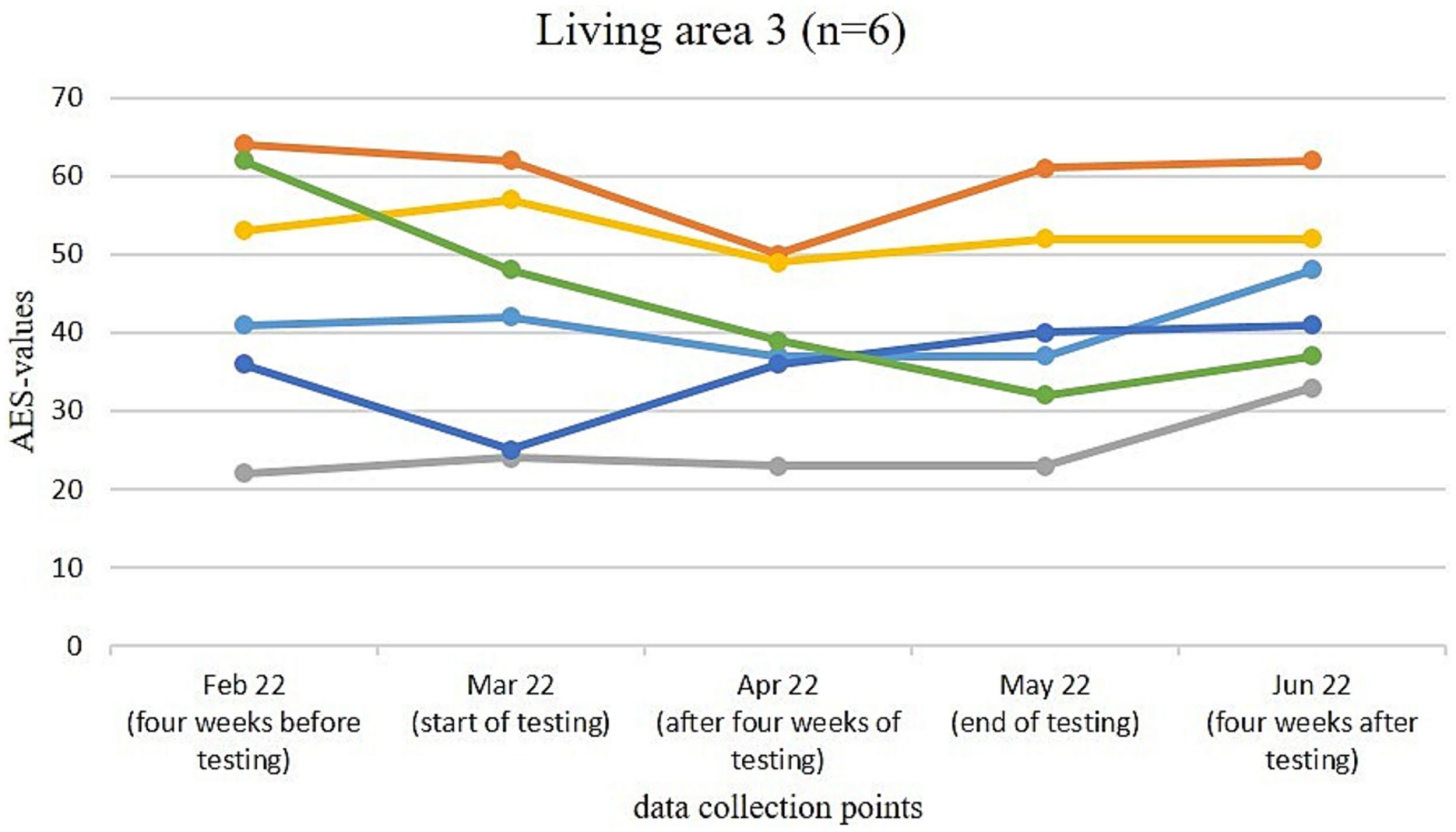
Change in the AES values of residents in living area 3.

**Figure 6:**
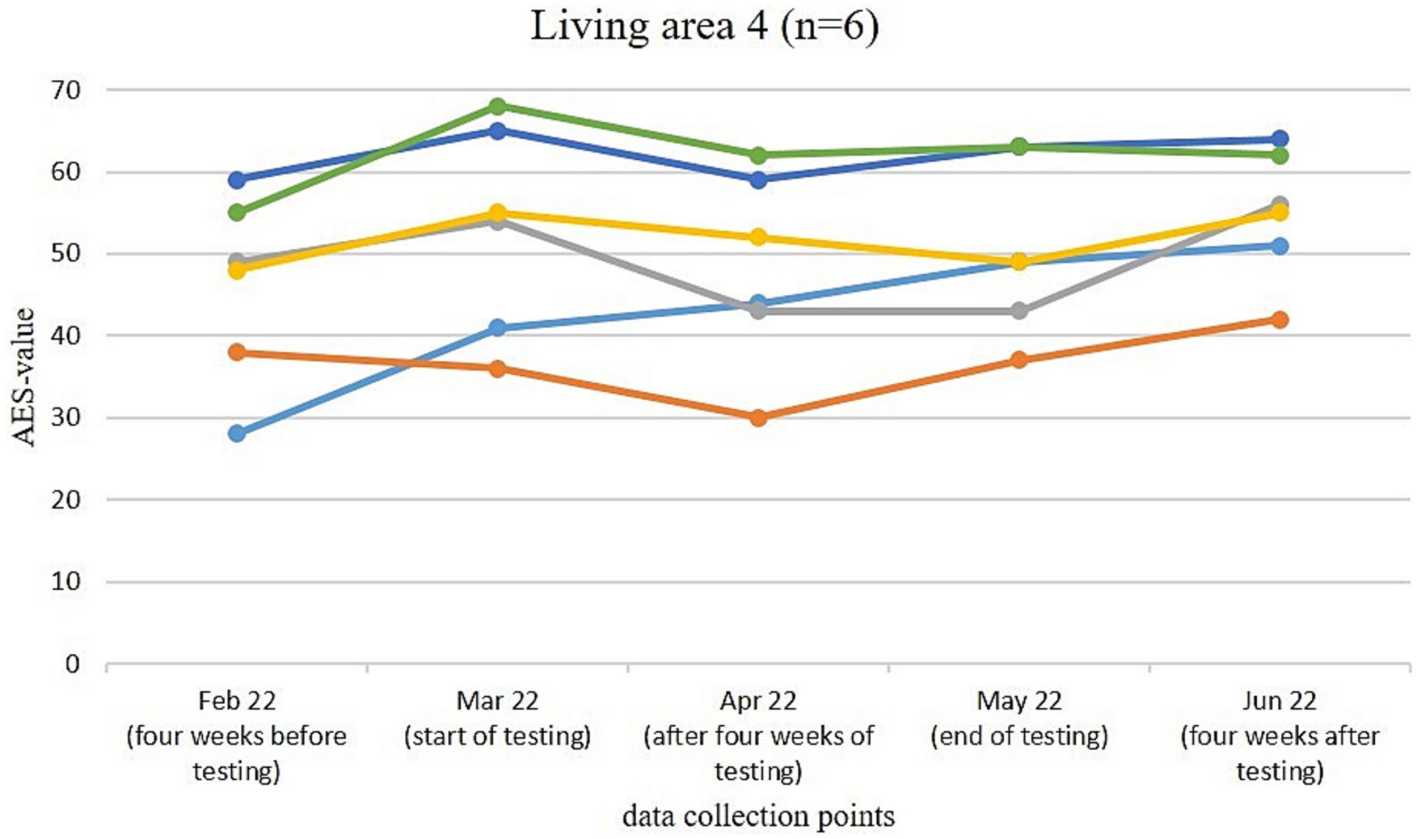
Change in the AES values of residents in living area 4.

Figures 3–6 illustrate the changes in apathy among the residents participating in the use of the Tovertafel^®^ in the four residential areas. One line always represents one person and shows the AES values at the five survey times, when the residents were recruited 4 weeks before the trial (February 2022), at the beginning of the trial (March 2022), during the trial after 4 weeks of using the Tovertafel^®^ (April 2022), at the end of the trial (May 2022) and 4 weeks after the trial (June 2022), and thus shows the change in apathy among the residents over time. AES values between 18 and 72 are possible, with higher values indicating higher levels of apathy.

In residential area 1, the apathy of residents ranged from 35 to 60 in February 2022. There was a reduction in apathy in four residents from February (4 weeks before the trial) to March 2022 (start of the trial) and also from March (start of Tovertafel^®^ use) to April 2022 (survey after 4 weeks of Tovertafel^®^ use). Five residents showed an increase in apathy at the end of the trial (May 2022) compared to the survey after 4 weeks of the Tovertafel^®^ trial (April 2022). Furthermore, five residents showed an increase in apathy from the end of the trial (May 2022) to the survey 4 weeks after the intervention (June 2022). The AES values in June 2022 were between 49 and 66 (Figure 3).

Figure 4 shows that the AES values of the residents in living area 2 were between 23 and 57 in February 2022. An increase in apathy is visible in all residents from February (4 weeks before the trial) to March 2022 (start of the trial). After the first 4 weeks of the Tovertafel^®^ trial (April 2022), three residents showed a reduction in their AES scores compared to the start of the trial (March 2022). In the following 4 weeks until May 2022 (end of the trial), five residents showed an increase in apathy compared to the survey after 4 weeks of Tovertafel^®^ intervention (April 2022). Apathy decreased in three residents from May (end of the trial) to June 2022 (4 weeks after the trial) and ranged between 20 and 68 in June 2022.

In residential area 3, the apathy of residents in February 2022 was between 22 and 64. From February (4 weeks before the trial) to March 2022 (start of the trial), the AES values fell for four residents. After the first 4 weeks of Tovertafel^®^ use (April 2022), five residents showed a reduction in apathy compared to the start of the trial (March 2022). From the survey after 4 weeks of Tovertafel^®^ use (April 2022) to the end of the intervention (May 2022), three residents showed a slight increase in apathy. There was also a slight increase in apathy in five residents from the end of the trial (May 2022) to 4 weeks after the trial (June 2022). The residents’ AES scores were between 33 and 62 4 weeks after the intervention in June 2022 (Figure 5).

The residents in living area 4 had AES values between 28 and 59 at the first survey in February 2022. Five residents showed an increase in apathy from February (4 weeks before the trial) to the start of Tovertafel^®^ use in March 2022. After 4 weeks of Tovertafel^®^ intervention (April 2022), five residents showed a reduction in apathy compared to the start of the trial (March 2022). From the survey after 4 weeks of Tovertafel^®^ use (April 2022) to the end of the trial (May 2022), a slight increase in apathy was observed in four residents. Also 4 weeks after the trial (June 2022), five residents showed a slight increase in AES values compared to the end of the intervention (May 2022). One month after the intervention in June 2022, the residents’ AES scores were between 42 and 64 (Figure 6).

Following the description of the changes in apathy in the study population, the changes in social activity (H3) will be presented on an ongoing basis. Two evaluations were carried out to show the changes in the overall group of study participants and according to the degree of dementia of the residents. In the following, the changes in the overall group of residents are discussed first, followed by the changes in residents with severe or moderate dementia.

### 3.4 Changes in social activity (H3)

#### 3.4.1 Positive expression (e.g., approaching, reaching out, touching, holding or grasping the activity, materials or people involved as a reaction to the activity)

The positive expression of behavioral participation was lowest in the overall group at observation time T1 (MW = 4.29; SD = 0.852) and increased at observation times T2 (MW = 4.30; SD = 1.125) and T3 (MW = 4.48; SD = 0.834) (Table 6).

**Table 6:** Positive expression of behavioral participation by degree of dementia and time of observation.

	Degree of dementia (MMST)	Mean value	Standard deviation	*n*
Behavioral participation (positive characteristic) – T1	Severe dementia	4.07	0.958	118
	Moderate dementia	4.50	0.678	126
	Total	4.29	0.852	244
Behavioral participation (positive characteristic) – T2	Severe dementia	4.07	1.175	118
	Moderate dementia	4.52	1.033	126
	Total	4.30	1.125	244
Behavioral participation (positive characteristic) – T3	Severe dementia	4.31	0.901	118
	Moderate dementia	4.64	0.732	126
	Total	4.48	0.834	244

A repeated-measures ANOVA with Huynh-Feldt correction found that the positive expression of behavioral involvement in the overall group differed statistically significantly between the observation times (*F*(1.864, 452.976) = 4.937; *p* = 0.009; partial *η*^2^ = 0.020). A post-hoc test showed a significantly higher positive expression of behavioral involvement at observation time T3 than at time points T1 (MW_Diff_ = −0.189; 95%-CI [0.052, 0.325]; *p* = 0.003) or T2 (MW_Diff_ = −0.176; 95%-CI [0.003, 0.350]; *p* = 0.045).

In participants with severe dementia, the positive expression of behavioral participation remained identical from observation time T1 (MW = 4.07; SD = 0.958) to time T2 (MW = 4.07; SD = 1.175) and increased at observation time T3 (MW = 4.31; SD = 0.901). For residents with moderate dementia, the positive expression of behavioral participation increased from observation time T1 (MW = 4.50; SD = 0.678) to time T2 (MW = 4.52; SD = 1.033) and was highest at observation time T3 (MW = 4.64; SD = 0.732) (Table 6).

A repeated-measures ANOVA with Huynh-Feldt correction showed that there was no significant interaction effect between the observation times and the MMST groups for the positive expression of behavioral involvement (*F*(1.871, 452.820) = 0.432; *p* = 0.636; partial *η*^2^ = 0.002).

The mean value of the positive expression of behavior-related participation at observation time T1 was 3.95 for residents with severe dementia, 4.44 for residents with moderate dementia and 4.21 for the overall group. The median value for the overall group and for residents with severe dementia was 4. At observation time T1, the standard deviation and variance were greatest for residents with severe dementia (SD = 1.044; *s*^2^ = 1.089) and greater in the overall group (SD = 0.943; *s*^2^ = 0.890) than for residents with moderate dementia (SD = 0.774; *s*^2^ = 0.598) (Table 7).

**Table 7:** Mean value comparison of the positive expression of behavior-related participation at time T1.

Degree of dementia (MMST)	Mean value	Median	Standard deviation	Variance (s^2^)
Severe dementia	3.95	4.00	1.044	1.089
Moderate dementia	4.44	5.00	0.774	0.598
Total	4.21	4.00	0.943	0.890

#### 3.4.2 Negative expression (e.g., avoidance, pushing away, withdrawal, hitting or inappropriate handling of the activity, the materials or the people involved as a reaction to the activity)

The negative expression of behavioral involvement was lowest in the overall group at observation time T1 (MW = 1.12; SD = 0.437), increased at time T2 (MW = 1.14; SD = 0.577) and returned to the initial value at observation time T3 (MW = 1.12; SD = 0.461) (Table 8).

**Table 8:** Negative expression of behavioral involvement by degree of dementia and time of observation.

	Degree of dementia (MMST)	Mean value	Standard deviation	*n*
Behavioral participation (negative characteristic) – T1	Severe dementia	1.21	0.583	118
	Moderate dementia	1.04	0.197	125
	Total	1.12	0.437	243
Behavioral participation (negative characteristic) – T2	Severe dementia	1.20	0.699	118
	Moderate dementia	1.07	0.425	125
	Total	1.14	0.577	243
Behavioral participation (negative characteristic) – T3	Severe dementia	1.15	0.517	118
	Moderate dementia	1.09	0.402	125
	Total	1.12	0.461	243

A repeated measures ANOVA with the assumption of sphericity showed that the negative expression of behavioral involvement did not differ significantly between the observation times (*F*(2, 484) = 0.085; *p* = 0.918; partial *η*^2^ = 0.000).

For residents with severe dementia, the negative expression of behavioral involvement decreased from observation time T1 (MW = 1.21; SD = 0.583) to time T2 (MW = 1.20; SD = 0.699) and to observation time T3 (MW = 1.15; SD = 0.517), in that order. In participants with moderate dementia, the negative expression of behavioral involvement increased from observation time T1 (MW = 1.04; SD = 0.197) to time T2 (MW = 1.07; SD = 0.425) and to observation time T3 (MW = 1.09; SD = 0.402) (Table 8).

A repeated measures ANOVA with the assumption of sphericity showed that there was no significant interaction effect between the observation times and the MMST groups with regard to the negative expression of behavioral involvement (*F*(2, 482) = 0.851; *p* = 0.427; partial *η*^2^ = 0.004).

The mean value of the negative expression of behavioral involvement at observation time T1 was 1.22 for residents with severe dementia, 1.13 for residents with moderate dementia and 1.17 for the overall group. The median in all groups was 1.00. The standard deviation and variance at observation time T1 were greatest for residents with severe dementia (SD = 0.607; *s*^2^ = 0.368) and greater in the overall group (SD = 0.563; *s*^2^ = 0.316) than for residents with moderate dementia (SD = 0.519; *s*^2^ = 0.269) (Table 9).

**Table 9:** Mean value comparison of the negative expression of behavior-related participation at time T1.

Degree of dementia (MMST)	Mean value	Median	Standard deviation	Variance (s^2^)
Severe dementia	1.22	1.00	0.607	0.368
Moderate dementia	1.13	1.00	0.519	0.269
Total	1.17	1.00	0.563	0.316

## 4 Discussion

The monocentric intervention study “Effects of the Tovertafel^®^ on apathy, social interaction and social activity of PWD through use in long-term inpatient care (Entertain),” based on the application context of *serious games for dementia care* (SGDC), was conducted and evaluated in the years 2021–2023. The study shows that the application and use of the Tovertafel^®^ in the context of care services has significant effects in some areas for people with moderate and severe dementia.

The questions addressed in the intervention study, such as how the use of the Tovertafel^®^ changes the social interaction or the extent of apathy of PWD in an inpatient long-term care facility, were examined and analyzed in the study. The answers to the research questions will now be elaborated in more detail.

### 4.1 Change in social interaction

Does the use of the Tovertafel^®^ change the social interaction of PWD in an inpatient long-term care facility (research question 1)?

The results are based on the assumption (see chapter 4.2) that significant results can be demonstrated by using the EPWDS scale in the implementation of the models using linear variance analysis with repeated measures. Accordingly, the use of the Tovertafel^®^ resulted in a positive change in social interaction. Using the Tovertafel^®^ resulted in a significantly higher positive social interaction during use than before playing with the Tovertafel^®^, a significantly lower positive social interaction one hour after using the Tovertafel^®^ than during playing and a significantly higher positive social interaction one hour after playing with the Tovertafel^®^ than before using it on the day of the game. However, there are tendencies toward learning effects with repeated use of the Tovertafel^®^. Negative social interaction, i.e., distracting or disturbing other residents, was significantly lower at the time of using the Tovertafel^®^ than before playing with the Tovertafel^®^. This illustrates that the Tovertafel^®^ has a positive effect on social interaction and that this effect persists for up to one hour after use. However, it has to be taken into account, that the survey took place during the COVID pandemic, a time when residents only had very limited contact with relatives and Tovertafel^®^ was the only social activity offered. Thus, the positive effects of the Tovertafel^®^ intervention might be overestimated compared to a setting without these restrictions.

### 4.2 Change of apathy

Furthermore, it was investigated how and whether the use of the Tovertafel^®^ changes the extent of apathy of PWD in an inpatient long-term care facility (research question 2).

The results suggest (see Chapter 4.3) that the use of the Tovertafel^®^ changes or temporarily reduces the apathy of the participating residents over the course of 2 months.

In all four living areas, there was mostly a reduction in apathy among residents after 4 weeks of Tovertafel^®^ use compared to the start of the intervention. At the end of the trial, however, there was an increase in apathy among the residents across all living areas compared to the apathy values during the intervention. One possible reason for this increase in apathy is the progression of the residents’ dementia.

In living areas 1, 3 and 4, 4 weeks after the end of the Tovertafel^®^ application, there was again an increase in resident apathy compared to the end of the intervention. Only in living area 2 are there tendencies that indicate that the positive effects of the Tovertafel^®^ on residents’ apathy have continued 4 weeks after the end of the intervention. One possible explanation is that only residents with moderate dementia took part in the intervention in residential area 2. However, it must be taken into account that 4 weeks after the end of the Tovertafel^®^ application, the residents of the four living areas usually had higher AES values and thus greater apathy than 4 weeks before the start of the intervention. As already described, this could be due to the progression of the residents’ dementia. The results thus make it clear that the use of the Tovertafel^®^ tends to have short-term positive effects on residents’ apathy, but that these do not tend to last in the long term.

### 4.3 Change in social activity

The study also investigated how the use of the Tovertafel^®^ changes the level of social activity of PWD in a long-term inpatient care facility.

The results show (see chapter 4.4) that social activity changes positively through the use of the Tovertafel^®^. However, it is important to note that the survey took place during the COVID pandemic, a time when residents had very limited contact with relatives and Tovertafel^®^ was the only social activity offered. Hence, the impact of social isolation and study related interaction in general must be taken into account when evaluating the results. One hour after using the Tovertafel^®^, a significantly higher level of positive social activity was observed than before and during playing with the Tovertafel^®^. This shows that the positive change in the level of social activity persists up to one hour after playing. The severity of the dementia (moderate or severe dementia) had no significant influence on the level of social activity when using the Tovertafel^®^. However, in the presence of severe dementia, there is a tendency toward learning effects with repeated use of the Tovertafel^®^. The results also show that negative social activity, such as pushing away or withdrawing, was slightly more pronounced during Tovertafel^®^ use than before use. However, since no negative effects on the well-being of the residents could be analyzed during the Tovertafel^®^ application, this can therefore be attributed less to the Tovertafel^®^ than to an imprecision in the measurement procedure.

### 4.4 Strengths of the study

One strength of the study can be seen in the fact that the testing took place in a real healthcare setting. By conducting the study under controlled conditions, an attempt was also made to minimize possible types of bias.

Another strength lies in the fact that during the use of the Tovertafel^®^, the implementation of social group activities was severely restricted due to the prevailing COVID-19 pandemic and only the use of the Tovertafel^®^ took place as a social group intervention in compliance with the protective measures (see chapter 3.3). For this reason, it is suggested that changes in apathy, social interaction, and social activity may be attributed primarily to the intervention with the Tovertafel^®^. However, given the non-controlled study design and the influence of group application, this conclusion must be interpreted with care.

As the trial took place with PWD as a vulnerable group, the sample size can be seen as a further strength. The test situation and the groups were homogeneous and there were hardly any participant dropouts during the course of the study. The survey period of several months is also a strength of the study, the structured implementation of which led to meaningful data. The survey instruments used can also be classified as valid and standardized questionnaires from the psychological field of social and behavioral sciences (MMST, AES, EPWDS) (9, 21, 22).

### 4.5 Limitations

Several limitations should be noted regarding this study.

First, there was no inclusion of a control group within the intervention study and the groups were not randomized, which leads to a limited significance of the analyzed results.

Second, the internal validity in this study can be seen as a limitation, compared to more elaborate and comprehensive approaches, e.g., the framework of the UK Medical Research Council for the development and evaluation of complex interventions (29). The limitation is due to the lean “within-subject” design of the study including the renunciation of control interventions. Further, the applied informant-based assessments are prone to some subjectivity bias. Video recordings, multiple independent raters or the usage of sensors might help to assess the observed symptoms more objectively. However, under the given pandemic conditions and circumstances, the chosen lean within-subject design without control interventions seemed to be a feasible approach to address the targeted research questions while taking into account the requirements of the care facility and following ethical considerations regarding the testing with a vulnerable target group.

As a third limitation it must be noted, that as the survey took place during the pandemic, the residents included in the study population only had very limited contact with relatives and social activity and social behavior of the residents was highly restricted. Hence, the positive effects of the Tovertafel^®^ intervention might be overestimated compared to a setting without these restrictions as the intervention was the only social intervention offered at that time.

Finally, the testing period in the residential areas was extended due to corona-related events in order to ensure a homogeneous test situation and comparability in the different residential areas. We are aware that the observers presence potentially influenced or disrupted the care situation or behavior of residents, however, the observers addressed this concern by approaching the residents empathetically and remaining in the background during the observation with no active involvement.

### 4.6 Prospective outlook

The study is based on the assumption that significant results can be recorded over time in people with moderate and severe dementia when using the Tovertafel^®^ over a period of 2 months. Significant results were found particularly in the areas of apathy, social activity and social interaction. It is a snapshot that statistically demonstrates that the use of the Tovertafel^®^ can have significant effects on people with moderate and severe dementia over a period of 2 months. In particular, it can reduce symptoms of apathy and increase social interaction and activity. The effects of the Tovertafel^®^ on apathy were still evident in individual residents 4 weeks after the end of the trial. Due to this possible reduction in residents’ apathy, regular use of the Tovertafel^®^ should be aimed for.

The study was able to evaluate the applicability of the Tovertafel^®^ for people with dementia in an inpatient setting. However, in order to be able to make further statements regarding the use of the Tovertafel^®^ and its long-term effects in people with dementia, further scientific studies are required that take place over a longer period of time and with defined and longer breaks between the Tovertafel^®^ applications, so that no carry-over effects (28) occur in the participants or these can at least be mitigated. Subsequent studies should also include a larger study population, be randomized controlled, include a control group and be implemented in a multicenter setting.

## Data Availability

The datasets presented in this article are not readily available because the permission to use datasets was only signed for internal purposes. Publication of datasets was excluded. Requests to access the datasets should be directed to Christian Bauer, christian.bauer@thws.de.

## Footnotes

^1^ DRKS-00027831
